# A nested cohort study of 6,248 early breast cancer patients treated in neoadjuvant and adjuvant chemotherapy trials investigating the prognostic value of chemotherapy-related toxicities

**DOI:** 10.1186/s12916-015-0547-5

**Published:** 2015-12-29

**Authors:** Jean E. Abraham, Louise Hiller, Leila Dorling, Anne-Laure Vallier, Janet Dunn, Sarah Bowden, Susan Ingle, Linda Jones, Richard Hardy, Christopher Twelves, Christopher J. Poole, Paul D P Pharoah, Carlos Caldas, Helena M. Earl

**Affiliations:** Department of Oncology, Addenbrooke’s Hospital, University of Cambridge, Hills Road, Box 193, Cambridge, CB2 0QQ UK; NIHR Cambridge Biomedical Research Centre and Cambridge Experimental Cancer Medicine Centre, Box 277, Hills Road, Cambridge, CB2 0QQ UK; Strangeways Research Laboratory, University of Cambridge, 2 Worts Causeway, Cambridge, CB1 8RN UK; Department of Oncology, Cambridge Cancer Trials Centre, Box 279 (S4), Addenbrooke’s Hospital, Cambridge, CB2 0QQ UK; Cambridge Breast Unit and Cambridge University Hospitals NHS Foundation Trust, Hills Road, Cambridge, CB2 0QQ UK; Warwick Clinical Trials Unit, University of Warwick, Gibbet Hill Road, Coventry, CV4 7AL UK; Cancer Research UK Clinical Trials Unit, Institute for Cancer Studies, University of Birmingham, Edgbaston, Birmingham, B15 2TT UK; Cancer Research UK Cambridge Institute, University of Cambridge, Li Ka Shing Centre, Robinson Way, Cambridge, CB2 0RE UK; Level 4, Leeds Institute of Cancer and Pathology and Leeds Experimental Cancer Medical Centre, St James Institute of Oncology, Beckett Street, Leeds, LS9 7TF UK

**Keywords:** Adverse events, Breast cancer, Chemotherapy, Prognosis, Survival, Toxicity

## Abstract

**Background:**

The relationship between chemotherapy-related toxicities and prognosis is unclear. Previous studies have examined the association of myelosuppression parameters or neuropathy with survival and reported conflicting results. This study aims to investigate 13 common chemotherapy toxicities and their association with relapse-free survival and breast cancer-specific survival.

**Methods:**

Chemotherapy-related toxicities were collected prospectively for 6,248 women with early-stage breast cancer from four randomised controlled trials (NEAT; BR9601; tAnGo; Neo-tAnGo). Cox proportional-hazards modelling was used to analyse the association between chemotherapy-related toxicities and both breast cancer-specific survival and relapse-free survival. Models included important prognostic factors and stratified by variables violating the proportional hazards assumption.

**Results:**

Multivariable analysis identified severe neutropenia (grades ≥3) as an independent predictor of relapse-free survival (hazard ratio (HR) = 0.86; 95 % confidence interval (CI), 0.76–0.97; *P* = 0.02). A similar trend was seen for breast cancer-specific survival (HR = 0.87; 95 % CI, 0.75–1.01; *P* = 0.06). Normal/low BMI patients experienced more severe neutropenia (*P* = 0.008) than patients with higher BMI. Patients with fatigue (grades ≥3) showed a trend towards reduced survival (breast cancer-specific survival: HR = 1.17; 95 % CI, 0.99–1.37; *P* = 0.06). In the NEAT/BR9601 sub-group analysis by treatment component, this effect was statistically significant (HR = 1.61; 95 % CI, 1.13–2.30; *P* = 0.009).

**Conclusions:**

This large study shows a significant association between chemotherapy-induced neutropenia and increased survival. It also identifies a strong relationship between low/normal BMI and increased incidence of severe neutropenia. It provides evidence to support the development of neutropenia-adapted clinical trials to investigate optimal dose calculation and its impact on clinical outcome. This is important in populations where obesity may lead to sub-optimal chemotherapy doses.

**Electronic supplementary material:**

The online version of this article (doi:10.1186/s12916-015-0547-5) contains supplementary material, which is available to authorized users.

## Background

Chemotherapy-related toxicities (CRTs) are a common complication of treatment in all cancers. For each CRT, multiple factors contribute to their development, including pharmacogenetic and co-morbidity factors [1]. The relationship between the occurrence of various CRTs and subsequent survival has been investigated in relatively small cohorts in multiple tumour types with conflicting results. A CRT may be a proxy pharmacokinetic parameter, indicating the level of drug exposure, dose density delivered and/or metabolic activity, or it may be a proxy pharmacodynamic parameter that reflects the sensitivity and susceptibility of different tissues to chemotherapy.

Many studies in different tumour types have investigated the association between survival and measures of myelosuppression. Eskander et al. [2] reviewed seven breast cancer studies with inter-study heterogeneity in trial design and varying toxicities, including leukocyte nadir, myelosuppression and neutropenia. The largest study [3] (n = 750), showed that patients with grade 2 or 3 neutropenia, on the National Cancer Institute Common Toxicity Criteria for Adverse Events (NCI CTCAE) scale, had a 10 % absolute survival advantage at 5 years compared to those with no neutropenia (multivariable *P* = 0.037). Shitara et al. [4] performed a meta-analysis of 13 trials (n = 9,528) considering several different toxicities, varying tumour types, stages of disease, and thresholds of NCI CTCAE classification and concluded that neutropenia or leukopenia experienced during chemotherapy was associated with improved survival.

The association between survival and taxane-related sensory neuropathy in breast cancer patients has been explored previously. Schneider et al. [5] investigated 4,554 patients from a randomised controlled clinical trial and found no significant relationship between neuropathy and disease-free survival (DFS), overall survival, or relapse-free survival (RFS). However, Moreno-Aspitia et al. [6] did report an association of taxane-related sensory neuropathy with DFS in early stage, taxane-treated, human epidermal growth factor (HER2)-positive breast cancer patients. In ovarian cancer, Lee et al. [7] found that sensory neuropathy secondary to treatment with paclitaxel and carboplatin was associated with improved progression-free survival (n = 949).

Moderate and/or severe oral mucositis was associated with improved survival in one study [8] (n = 533). Another study associated oral mucositis with an increased risk of infection and an adverse impact on survival [9].

Although there is considerable data on the impact of fatigue on quality of life [10, 11] in early stage breast cancer, there is no published evidence on the prognostic significance of chemotherapy-induced fatigue in early stage disease.

We have investigated the association between 13 common CRTs and RFS and breast cancer-specific survival (BCSS) in patients (n = 6,248) with early stage breast cancer using data from randomised controlled trials with prospective protocol-driven collection of CRTs.

## Methods

### Patients and clinical trials

Clinical data was collected from the UK randomised clinical trials NEAT (n = 2027) [12], BR9601 (n = 374) [12], tAnGo (n = 3152) [13], and Neo-tAnGo (n = 831) [14], creating a nested cohort of 6,248 patients, from a total of 6,384 patients, included in this study after providing adequate quality toxicity data. Additional file 1: Figure S1a,b summarises the individual clinical trials included and their trial objectives. Table 1 summarises patient characteristics of the 6,248 patients, with Additional file 1: Table S1 showing patient characteristics by each trial. Median follow-up was 6.2 years, with 1,335 (21 %) breast cancer-related events, 148 (2 %) non-breast cancer-related deaths, and 4,765 (77 %) live patients. For the analysis of RFS, there were 1,888 events (30 %) recorded and 4,360 (70 %) censored observations. Written informed consent was obtained from each patient recruited into the trials. All the trials involved received full ethical approval from a UK ethical review board and completed all other regulatory requirements prior to commencement.

**Table 1:** Summary of patient characteristics for the study cohort

	Study cohort	
	n	%
Randomised treatment		
E-CMF	1156	19
CMF	1149	19
EC-T	1773	28
EC-TG	1769	28
T-EC	200	3
TG-EC	201	3
Age, years		
≤ 50	3606	58
> 50	2642	42
ER status		
Negative	2551	41
Positive	3591	57
Missing	106	2
pGR status		
Negative	2463	39
Positive	2652	43
Missing	1133	18
HER2 status		
Negative	3760	60
Positive	1034	17
Missing	1454	23
Nodal status		
Negative	1366	22
1–3 positive	2383	38
Clinically negative, neoadjuvant	409	7
Clinically positive, neoadjuvant	403	6
4+ positive	1687	27
Breast cancer-specific survival		
Breast cancer related deaths	1335	21
Deaths due to other cause	148	2
Alive	4765	77
Relapse-free survival		
Events	1888	30
Censored	4360	70
Triple negative status		
No (ER^+^ and HER2^–^)	2321	37
Yes (ER^–^, PGR^–^ or unknown, and HER2^–^)	1242	20
Missing	2685	43
ECOG performance status		
0	5232	84
≥ 1	683	11
Missing	333	5
Tumour size, mm		
0–20	2195	35
21–50	3375	54
> 50	475	8
Missing	203	3
Tumour grade		
1	146	2
2	2206	35
3	3654	59
Missing	242	4
Menopausal status		
Pre/peri	3480	56
Post	2200	35
Missing	568	9
BMI		
Underweight (<18.5)	69	1
Healthy weight (18.5 to <25)	2503	40
Overweight (25 to <30)	2088	33
Obese (≥30)	1469	24

*ER* Estrogen receptor; *pGR* Progesterone receptor; *HER2* Human epidermal growth factor receptor; *ECOG* Eastern Co-operative Oncology Group; *BMI* Body mass index; *E* Epirubicin; *C* Cyclophosphamide; *M* Methotrexate; *F* 5-fluouroucil; *T* Paclitaxel; *G* Gemcitabine

### Phenotypes

**Fig. 1:**
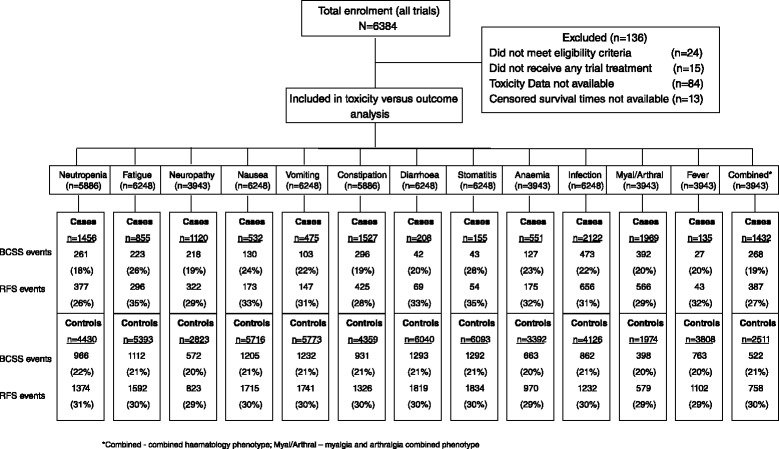
Consort diagram for chemotherapy-related toxicity analyses. *Combined, combined haematology phenotype; Myal/Arthral, myalgia and arthralgia combined phenotype

In all trials, CRTs were evaluated during each chemotherapy cycle for each patient. CRTs were graded using NCI CTCAE (version 2 or 3; Table S2 in Additional file 1) by the investigators at the participating centres and data collected centrally via case report forms. For each of the 13 CRTs of interest (Fig. 1 - Consort diagram for chemotherapy-related toxicity analyses), patients were categorised into a case or control based on their maximum reported grade of the CRT throughout their chemotherapy treatment (Table S3, Additional file 1). All trials required pre-treatment blood count assessment prior to administration of each cycle and neutropenia was classified from immediate pre-chemotherapy blood tests. Blood draw was avoided during the expected white blood cell nadir period.

### Ethics, consent and permissions

Ethical approval was obtained for tAnGo (West Midlands: 00/7/44), Neo-tAnGo (South East: 04/MRE01/60), NEAT/BR9601 (West Midlands: 30/04/1996) and PGSNPS (Cambridgeshire: 05Q0108/71).

All patients gave their consent to participate in the trials.

### Statistical analysis

To investigate the association between CRTs and outcomes, BCSS time was calculated from date of treatment cessation to date of death due to breast cancer, or to date of death due to other causes, or date of censoring in women still alive. RFS time was calculated from date of treatment cessation to either the date of first relapse or date of death in women dying without relapse, or to date of censoring for those alive and relapse free.

Cox proportional-hazards modelling was used to investigate the association between CRTs experienced (categorised as shown in Additional file 1: Table S3) and BCSS and RFS. A base Cox model was created by testing the association of important prognostic factors with BCSS and RFS. Any factors which were significant at *P* <0.05 in univariable analysis were entered into a multivariable Cox model and factors remaining significant on adjustment in the multivariable model were retained for the base Cox model. The proportional hazards assumption was checked using the Schoenfeld residuals method [15]. Subsequent models were stratified by variables that violated the proportional hazards assumption. All CRTs significant in a univariable Cox model at the *P* <0.1 level were entered into the multivariable base Cox model to assess their association with BCSS and RFS. Associations between CRTs and BCSS or RFS were deemed statistically significant if the *P* value was <0.05.

Any CRT showing a relationship with outcome at this stage was further investigated. To determine if the relationship found was independent of dose intensity (DI) (sub-optimal DI (<85 %) versus optimal DI (>85 %)) [16], the analysis was re-run adjusting for DI. Due to the known relationship between increasing BMI and poor prognosis [17, 18], we similarly assessed if adjusting for BMI affected the relationship between the CRT and RFS/BCSS. Additionally, CRT relationships with known prognostic factors were assessed using χ^2^ tests with continuity corrections.

After performing this analysis on all 6,248 patients, seven different components of the treatment regimens received by the group of patients were investigated (Table S4 in Additional file 1), including (1) epirubicin (E); (2) cyclophosphamide, methotrexate and 5-fluorouracil (CMF) after having received E; (3) CMF as the sole treatment regimen; (4) EC as a primary component; (5) paclitaxel (T) and/or gemcitabine (G) after receiving EC; (6) T and/or G as a primary component; and (7) EC after having received T and/or G). Case-control re-classification for each of the CRTs of interest was undertaken focusing purely on the patients’ maximum reported grade during the different components of their particular treatment regimen. Association with increased or decreased RFS and BCSS was assessed.

## Results

### Analysis of maximum CRT across all chemotherapy treatments

The base Cox model included trial, performance status (PS) and nodal status, and was stratified by tumour size, tumour grade and estrogen receptor (ER) status. Neutropenia, fatigue, anaemia, combined haematological toxicity and constipation were nominally significant (*P* <0.1) for either BCSS or RFS (or both) on univariable analysis (Table 2). All other CRTs were not found to be associated with either RFS or BCSS. After adjustment for other prognostic factors in the model, only neutropenia was significant. Fatigue was not statistically significant after adjustment (BCSS; HR = 1.17; 95 % CI, 0.99–1.37; *P* = 0.06).

**Table 2:** Analysis of maximum CRT across all treatments

Toxicity	n (Univariable analysis)	n (Multivariable analysis)	BCSS				RFS			
			Unadjusted		Adjusted		Unadjusted		Adjusted	
			HR (95 % CI)	*P* value	HR (95 % CI)	*P* value	HR (95 % CI)	*P* value	HR (95 % CI)	*P* value
Neutropenia^a^	5886	5211	0.85 (0.74–0.98)	0.02	0.87 (0.75–1.01)	0.06	0.85 (0.76–0.95)	0.004	0.86 (0.76–0.97)	0.02
Nausea	6248	5468	1.11 (0.93–1.33)	0.25	1.09 (0.90–1.33)	0.37	1.07 (0.91–1.25)	0.42	1.06 (0.90–1.26)	0.47
Vomiting	6248	5468	0.98 (0.80–1.19)	0.80	1.03 (0.84–1.28)	0.76	1.00 (0.85–1.18)	>0.99	1.05 (0.88–1.26)	0.57
Stomatitis	6248	5468	1.20 (0.89–1.63)	0.24	1.25 (0.90–1.74)	0.19	1.09 (0.83–1.43)	0.54	1.12 (0.83–1.50)	0.46
Constipation	5886	5211	0.91 (0.80–1.04)	0.17	0.95 (0.82–1.09)	0.45	0.91 (0.82–1.01)	0.09	0.94 (0.83–1.05)	0.27
Diarrhoea	6248	5468	0.93 (0.68–1.26)	0.64	0.98 (0.70–1.38)	0.93	1.08 (0.85–1.37)	0.54	1.19 (0.92–1.55)	0.18
Infection	6248	5468	1.09 (0.97–1.22)	0.14	1.01 (0.90–1.15)	0.82	1.06 (0.96–1.16)	0.26	1.01 (0.91–1.12)	0.88
Fatigue	6248	5468	1.24 (1.07–1.43)	0.004	1.17 (0.99–1.37)	0.06	1.17 (1.03–1.32)	0.01	1.13 (0.99–1.30)	0.08
Anaemia	3943	3582	1.18 (0.97–1.42)	0.09	1.14 (0.93–1.39)	0.21	1.11 (0.95–1.30)	0.20	1.08 (0.91–1.28)	0.36
Combined haematological	3943	3582	0.89 (0.77–1.03)	0.11	0.88 (0.76–1.03)	0.12	0.88 (0.78–0.99)	0.03	0.88 (0.78–1.00)	0.06
Neurotoxicity	3943	3582	0.96 (0.82–1.13)	0.64	0.99 (0.84–1.17)	0.90	0.98 (0.86–1.12)	0.78	0.99 (0.87–1.14)	0.94
Myalgia	3943	3582	0.95 (0.82–1.09)	0.43	1.03 (0.89–1.20)	0.69	0.95 (0.84–1.06)	0.34	1.02 (0.91–1.16)	0.70
Fever	3943	3582	0.98 (0.67–1.44)	0.91	0.84 (0.57–1.26)	0.41	1.08 (0.80–1.46)	0.63	0.98 (0.71–1.34)	0.88

*BCSS* Breast cancer-specific survival; *RFS* Relapse-free survival; *HR* Hazard ratio; *CI* Confidence interval

^a^Cases classified as National Cancer Institute Common Toxicity Criteria for Adverse Events (NCI CTCAEAE) grade ≥3

#### Neutropenia

Neutropenia status was available for 5,886 patients, of whom 1,456 (25 %) recorded neutropenia grade ≥3 over the course of their entire chemotherapy treatment; 4,430 (75 %) did not. After adjusting for the base model prognostic factors, neutropenia remained a statistically significant, independent predictor of RFS (HR = 0.86; 95 % CI, 0.76–0.97; *P* = 0.02), with a similar trend seen for BCSS (HR = 0.87; 95 % CI, 0.75–1.01; *P* = 0.06). The association was strengthened after further adjustment for DI (BCSS: HR = 0.85; 95 % CI, 0.73–0.98; *P* = 0.03; RFS: HR = 0.84; 95 % CI, 0.74–0.95; *P* = 0.005). Patients with grade ≥3 neutropenia were likely to survive and remain relapse free for longer when compared to patients who experienced grades ≤2. Neutropenia appeared unrelated to triple negative status (*P* = 0.85), ER status (*P* = 0.46), and HER2 status (*P* = 0.36).

Underweight and normal BMI patients were more likely to record severe neutropenia during their treatment course than overweight or obese BMI patients (27 % vs. 24 %, *P* = 0.008). Given the known association of increasing BMI with poor prognosis [17, 18], we repeated the analysis adjusting for BMI, to confirm that the relationship between neutropenia and RFS/BCSS was independent of BMI. Adjusting for BMI only marginally changed the results (BCSS: HR = 0.88; 95 % CI, 0.76–1.02; *P* = 0.08; RFS: HR = 0.87; 95 % CI, 0.77–0.98; *P* = 0.02). Adjustment for both DI and BMI simultaneously resulted in similar findings to when adjusting only for DI (BCSS: HR = 0.85; 95 % CI, 0.74–0.99; *P* = 0.04; RFS: HR = 0.84; 95 % CI, 0.74–0.95; *P* = 0.006). Underweight and normal patients were also more likely to report moderate-severe vomiting (grade ≥2, *P* = 0.009). Obese and overweight patients recorded more diarrhoea (grade ≥2, *P* = 0.02), infection (grade ≥2, *P* = 0.005), neuropathy (grade ≥2, *P* < 0.0001) and arthralgia/myalgia (grade ≥2, *P* = 0.002), but these CRTs were not associated with either improved or reduced survival.

Older patients and post-menopausal patients were more likely to have neutropenia during their treatment course (28 % vs. 22 %, *P* <0.0001 and 28 % vs. 22 %, *P* <0.0001, respectively).

The extent of neutropenia experienced by the study cohort was not influenced by the use of prophylactic growth colony stimulating factor (GCSF). GCSF was not routinely given as prophylaxis as part of the trial protocol of any of the study trials. GCSF use was allowed secondary to an admission for febrile neutropenia and/or based on clinical judgement. However, only in 9 % of patients was the use of GCSF ever reported. It is unlikely, therefore, that this would have a significant impact on the overall results shown.

As previous studies [19] have classified neutropenia as no neutropenia versus any (grade 0 vs. ≥1), we repeated the neutropenia analysis using this classification (Table 3). The multivariable analysis demonstrated a statistically significant association between neutropenia and BCSS (HR = 0.87; 95 % CI, 0.77–0.99; *P* = 0.03) with a similar trend for RFS (HR = 0.91; 95 % CI, 0.82–1.01; *P* = 0.07).

**Table 3:** Analysis of neutropenia across all treatments (classification National Cancer Institute Common Toxicity Criteria for Adverse Events (NCI CTCAE) grade ≥1 vs. grade 0)

Toxicity	n (univariable analysis)	n (multivariable analysis)	BCSS				RFS			
			Unadjusted		Adjusted		Unadjusted		Adjusted	
			HR (95 % CI)	*P* value	HR (95 % CI)	*P* value	HR (95 % CI)	*P* value	HR (95 % CI)	*P* value
Neutropenia	5,886	5,211	0.86 (0.77–0.96)	0.009	0.87 (0.77–0.99)	0.03	0.91 (0.83–0.99)	0.04	0.91 (0.82–1.01)	0.07

*BCSS* Breast cancer-specific survival; *RFS* Relapse-free survival; *HR* Hazard ratio; *CI*, Confidence interval

#### Fatigue

Fatigue status was available for 6,248 patients, of whom 855 (14 %) recorded fatigue grades ≥3 at some point in their chemotherapy treatment whereas 5,393 (86 %) did not. Fatigue was associated with poorer BCSS and RFS in the univariable models, but the associations were attenuated and no longer significant after adjusting for other prognostic variables (BCSS: HR = 1.17; 95 % CI, 0.99–1.37; *P* = 0.06; RFS: HR = 1.13; 95 % CI, 0.99–1.30; *P* = 0.08).

Fatigue appeared unrelated to BMI (*P* = 0.76), triple negative status (*P* = 0.50), HER2 status (*P* = 0.86), age (*P* = 0.33), menopausal status (*P* = 0.27), and GCSF administration (*P* = 0.89). ER negative patients may be more likely to be classed as a fatigue case during their treatment course (15 % vs. 13 % of ER positive patients, *P* = 0.06), although this was not statistically significant at the *P* = 0.05 threshold and may not be of clinical significance.

### CRTs of interest during specific chemotherapy regimen components

In order to identify if CRTs reported during particular combination chemotherapy treatments were associated with increased or decreased RFS and BCSS, the analysis was repeated considering only the maximum NCI CTCAE grade documented during a specific chemotherapy regimen, rather the maximum NCI CTCAE grade across all chemotherapy treatments (Additional file 1: Table S4).

#### Neutropenia

Patients who experienced neutropenia grade ≥3 whilst receiving epirubicin and cyclophosphamide (EC) as their first chemotherapy component (drug regimen 4) were significantly more likely to survive and remain relapse free than those who did not (BCSS: HR = 0.83; 95 % CI, 0.69–1.00; *P* = 0.05; RFS: HR = 0.85; 95 % CI, 0.73–0.99; *P* = 0.04) (Additional file 1: Table S4). However, experiencing neutropenia grade ≥3 whilst receiving T ± G as their second chemotherapy component (having already received EC, drug regimen 5) was not significantly associated with outcome (BCSS: HR = 1.07; 95 % CI, 0.83–1.37; *P* = 0.62; RFS: HR = 1.00; 95 % CI, 0.80–1.24; *P* = 0.99).

Neutropenia grade ≥3 being reported either during T ± G as a first chemotherapy component, or during EC as the second chemotherapy component (after T ± G) was not significantly associated with outcome (drug regimens 6 and 7), although the numbers of patients in these datasets were small (n = 270 and 260, respectively).

Furthermore, when drug regimens 4 (CRT recorded in EC, as the first chemotherapy) and 7 (CRT recorded in EC, as the second chemotherapy after T ± G) were combined to create a sample consisting of all tAnGo and Neo-tAnGo patients receiving EC at any cycle of their treatment, severe neutropenia was significantly associated with both increased BCSS (HR = 0.83; 95 % CI, 0.69–0.99; *P* = 0.04) and RFS (HR = 0.84; 95 % CI, 0.73–0.98; *P* = 0.03). Neutropenia was not a significant predictor of survival for tAnGo and Neo-tAnGo patients when it occurred during T ± G regimens.

#### Fatigue

Patients experiencing moderate-severe fatigue whilst receiving epirubicin (E) as their first chemotherapy component (drug regimen 1) had significantly worse outcomes (BCSS: HR = 1.48; 95 % CI, 1.03–2.12; *P* = 0.03; RFS: HR = 1.34; 95 % CI, 0.97–1.85); *P* = 0.07) than those who did not record moderate-severe fatigue. Similarly, patients reporting severe fatigue during the period they received CMF as their second chemotherapy component (after completing E; drug regimen 2), had reduced BCSS (HR = 1.61; 95 % CI, 1.13–2.30; *P* = 0.009) and RFS (HR = 1.39; 95 % CI, 1.01–1.92; *P* = 0.05). Interestingly, this strong effect was not seen for NEAT and BR9601 patients receiving CMF only (drug regimen 3). Fatigue was not a significant predictor of survival for tAnGo and Neo-tAnGo patients on EC and T ± G (Additional file 1: Table S4: drug regimens 4–7).

## Discussion

We have investigated the association between 13 CRTs and clinical outcome (RFS and BCSS) in 6,248 patients with early breast cancer treated within randomised clinical trials of neoadjuvant and adjuvant chemotherapy. The majority of CRTs were not associated with either RFS or BCSS. However, we have demonstrated that severe neutropenia (grades ≥3) is associated with improved RFS. In addition, after re-classification of neutropenia case status to grades ≥1, the association with BCSS remains. Previous studies investigating the relationship between neutropenia and survival have hypothesised that neutropenia is a reflection of chemotherapy efficacy and activity. This implies that patients who are not achieving neutropenia may also not be receiving an effective or adequately active dose [18]. It must be noted that, in this study, 13 separate CRTs have been tested against two clinical endpoints of BCSS and RFS and, as such, the study findings must be considered whilst bearing in mind the issue of multiple testing.

Patients with normal or underweight BMI are more likely to have severe neutropenia during their treatment course in comparison to overweight or obese BMI patients (*P* = 0.008). In obese patients, due to an increase in the amount of fat contributing to the actual weight and potentially changes in blood flow, the pharmacokinetics of chemotherapy may be affected. This may affect volume of distribution, clearance, and, consequently, patient drug exposure. Thus, overweight or obese BMI patients may not be receiving an adequate dose, although further investigations would be required to confirm this. The disadvantages of using body surface area (BSA) to dose patients have been discussed at length elsewhere [20–22]. Drug disposition can show 4–10-fold inter-individual variability, which is inadequately compensated for by using BSA. There is a strong argument to include other variables to allow more accurate dose estimation for each individual.

Bergh et al. [23] conducted a randomised trial comparing high-dose chemotherapy versus haematologically-tailored adjuvant chemotherapy to assess the effect on RFS and overall survival. The haematologically-tailored arm specified that participants experienced pre-defined levels of haematological toxicities. This study demonstrated that tailored chemotherapy rather than high-dose chemotherapy showed improved RFS. One shortcoming of this study was that it used high-dose chemotherapy as the ‘control’ arm, which is not the current standard treatment for early breast cancer. Lindeman et al. [24] conducted a trial using haematological criteria for selecting dosing strategy. Study results, available in abstract form only, did not show a statistically significant improvement in survival with tailored-chemotherapy compared with standard BSA-based chemotherapy. However, both distant DFS and DFS showed trends towards a better outcome for the tailored chemotherapy arm. Our results show that chemotherapy-induced neutropenia is an additional prognostic factor for longer-term outcomes, and we suggest that this could be tested in trials randomising between personalising chemotherapy within an adaptive protocol and standard chemotherapy dosing. Interestingly, trials in which doses of cyclophosphamide and doxorubicin were increased (NSABP B22 [25], NSABP B25 [26], and CALGB 9344 [27]) did not show benefit in long-term outcomes. However, dose-dense trials which increased the frequency of chemotherapy dosing with filgrastim support (CALGB 9741 [28] and a 10-study meta-analysis [29]) have shown improvement in longer-term outcomes. The authors of CALGB 9741 stated that it was their impression that the improvements seen in CALGB 9741 were as a result of the more frequent administration of chemotherapy, and that use of filgrastim did not by itself add to the efficacy of dose-dense treatment [30].

These trials have applied dose intensification with filgrastim support, which has abrogated any ‘signal’ from neutropenia; in fact, they have usually shown less febrile neutropenia and neutropenic sepsis in the dose-dense arm. In our study, we have looked at the prognostic effect of developing neutropenia in trials which have used standard chemotherapy dosing without routine filgrastim support. Future trials would need to establish whether adapting doses to achieve neutropenia would improve outcomes. Our hypothesis is that host factors (such as pharmacodynamic and pharmacogenomic factors) influence the level of chemotherapy-induced neutropenia, and only those patients who do not achieve neutropenia with standard doses may benefit from intensification of chemotherapy. The evidence would suggest that adapting to a dose-dense protocol may be the most effective way to intensify chemotherapy. The results show that patients achieving grade ≥1 have a statistically significant survival advantage. This allows the possibility of using a simple standard haematological measurement to adjust dose, whereby dose is adjusted until neutropenia NCI CTCAE grade ≥1 is achieved, after which treatment would be maintained at the same dosing level.

The potential mechanisms that may explain why chemotherapy-induced neutropenia is associated with improved survival include neutropenia as a marker of cancer stem cell death [22, 31]. Other studies have proposed that neutrophils may be involved in the control of the microenvironment in sites of metastatic spread [32–34]. Chia et al. [35] comment that toxicity and clinical outcome may be more likely to correlate when the therapeutic agent targets the biological driver of the disease directly, for example, sunitinib-associated hypertension [36]. Although the mechanism behind the association between chemotherapy-induced neutropenia and clinical outcome is unclear, this study, in conjunction with previously published data [2-4], provides strong evidence that this association is real and clinically relevant.

Moderate-severe fatigue (NCI CTCAE ≥2) may be associated with a reduced BCSS. This effect was significant in the univariable analysis of fatigue across all treatments (HR = 1.24; 95 % CI, 1.07–1.43; *P* = 0.004) but on multivariable analysis BCSS (HR = 1.17; 95 % CI, 0.99–1.37; *P* = 0.06) became less significant. However, analysis of the treatment regimens showed that there was a statistically significant association between moderate-severe fatigue and BCSS in patients treated with ECMF (Additional file 1: Table S4). The patient characteristics across all the trials is similar, although 20 % of patients in NEAT and BR9601 had fatigue classified as grade ≥2, in comparison to only 10 % in tAnGo and Neo-tAnGo. It is notable that 18 % of NEAT and BR9601 had a pre-treatment PS ≥1, in comparison to only 8 % and 4 % of tAnGo and Neo-tAnGo patients, respectively (Additional file 1: Table S1). Trial eligibility criteria required that patients must have a PS ≤2. It is unclear whether this difference in baseline PS alone accounts for the increased levels of moderate-severe fatigue seen in NEAT and BR9601; however, reduced pre-treatment PS would be likely to increase the risk of severe fatigue during treatment [10, 11].

## Conclusions

This large and comprehensive study has shown a statistically significant association between improved survival and neutropenia (using toxicity classification NCI CTCAE ≥1 or ≥3). This association is clinically relevant and has the potential to be further tested in neutropenia-adapted treatment regimens within clinical trials to assess its potential to improve clinical outcome. This study shows that patients with normal or reduced BMI experience greater rates of neutropenia in comparison to overweight and obese patients. This is particularly relevant in populations where increasing levels of obesity may mean that a significant proportion of breast cancer patients are receiving sub-optimal chemotherapy doses. This study also indicates that chemotherapy-induced fatigue may be an indicator of poor clinical outcome. Patients’ pre-treatment performance status needs to be adequately assessed and levels of treatment-induced fatigue need to be carefully monitored and moderated.

## Availability of data and materials

Data supporting these findings is held by the Trial Management Group for PGSNPS study, where the original concept for this analysis was designed. Any access requires appropriate ethical approvals and would be assessed by the Trial Management Group which includes the respective Chief Investigators of the clinical trials and PGSNPS, and would require a specific Data Transfer Agreement.

## Additional file

Additional file 1: Table S1. Patient characteristics of the 6,248 patients shown by trial. **Table S2.** National Cancer Institute Common Toxicity Criteria for Adverse Events (NCI CTCAE) version 2. **Table S3.** Case-control classification method for the 13 CRTs investigated. **Table S4.** Neutropenia and fatigue in relation to outcome, split by different treatment components. **Figure S1.** Trial objectives, outcomes and treatment regimens of the contributing clinical trials. (DOCX 82 kb)
