# Supplementary material for: A nested cohort study of 6,248 early breast cancer patients treated in neoadjuvant and adjuvant chemotherapy trials investigating the prognostic value of chemotherapy-related toxicities
Source: BMC Med. 2015 Dec 29;13:306. doi: 10.1186/s12916-015-0547-5 (PMC4693418; doi:10.1186/s12916-015-0547-5)
Supplement: Additional file 1: Table S1. — Patient characteristics of the 6,248 patients shown by trial. Table S2. National Cancer Institute Common Toxicity Criteria for Adverse Events (NCI CTCAE) version 2. Table S3. Case-control classification method for the 13 CRTs investigated. Table S4. Neutropenia and fatigue in relation to outcome, split by different treatment components. Figure S1. Trial objectives, outcomes and treatment regimens of the contributing clinical trials. (DOCX 82 kb) [file 12916_2015_547_MOESM1_ESM.docx]

**SupplementaryTables &Figures**

Table S1: Patient characteristics of the 6248 patients shown by trial

Table S2: NCI CTCAE version 2

Table S3: Case-Control Classification method for the 13 CRTs investigated

Table S4: Neutropenia and Fatigue in relation to outcome, split by different treatment components

Figure S1a & b: Trial Objectives, outcomes and treatment regimens of the contributing Clinical Trials

**Table S1: Patient characteristics of the 6248 patients shown by trial**

|  | **NEAT/BR9601 (n=2305)** | | **tAnGo (n=3131)** | | **Neo-tAnGo (n=812)** | |
| --- | --- | --- | --- | --- | --- | --- |
|  | **N** | **%** | **N** | **%** | **N** | **%** |
| **Randomised Treatment** |  |  |  |  |  |  |
| **E-CMF** | 1156 | 50 | - | - | - | - |
| **CMF** | 1149 | 50 | - | - | - | - |
| **EC-T** | - | - | 1566 | 50 | 207 | 25 |
| **EC-TG** | - | - | 1565 | 50 | 204 | 25 |
| **T-EC** | - | - | - | - | 200 | 25 |
| **TG-EC** | - | - | - | - | 201 | 25 |
|  |  |  |  |  |  |  |
| **Age** |  |  |  |  |  |  |
| **≤50** | 1369 | 59 | 1725 | 55 | 512 | 63 |
| **>50** | 936 | 41 | 1406 | 45 | 300 | 37 |
|  |  |  |  |  |  |  |
| **ER Status** |  |  |  |  |  |  |
| **negative** | 904 | 39 | 1376 | 44 | 271 | 33 |
| **positive** | 1295 | 56 | 1755 | 56 | 541 | 67 |
| **missing** | 106 | 5 | - | - | - | - |
|  |  |  |  |  |  |  |
| **pGR Status** |  |  |  |  |  |  |
| **negative** | 769 | 33 | 1395 | 45 | 299 | 37 |
| **positive** | 968 | 42 | 1376 | 44 | 308 | 38 |
| **missing** | 568 | 25 | 360 | 11 | 205 | 25 |
|  |  |  |  |  |  |  |
| **HER2 Status** |  |  |  |  |  |  |
| **negative** | 1468 | 64 | 1790 | 57 | 502 | 62 |
| **positive** | 373 | 16 | 474 | 15 | 187 | 23 |
| **missing** | 464 | 20 | 867 | 28 | 123 | 15 |
|  |  |  |  |  |  |  |
| **Nodal Status** |  |  |  |  |  |  |
| **negative** | 641 | 28 | 725 | 23 | - | - |
| **1-3 positive** | 1092 | 47 | 1291 | 41 | - | - |
| **4+ positive** | 572 | 25 | 1115 | 36 | - | - |
| **clinically negative, neoadjuvant** | - | - | - | - | 409 | 50 |
| **clinically positive, neoadjuvant** | - | - | - | - | 403 | 50 |
|  |  |  |  |  |  |  |
| **Triple Negative Status** |  |  |  |  |  |  |
| **No (ER pos and HER2 neg)** | 919 | 40 | 1059 | 34 | 343 | 42 |
| **Yes (ER neg, PGR neg or UK and HER2 neg)** | 446 | 19 | 650 | 21 | 146 | 18 |
| **missing** | 940 | 41 | 1422 | 45 | 323 | 40 |
|  |  |  |  |  |  |  |
| **ECOG performance status** |  |  |  |  |  |  |
| **0** | 1617 | 70 | 2889 | 92 | 726 | 89 |
| **≥1** | 411 | 18 | 242 | 8 | 30 | 4 |
| **missing** | 277 | 12 | - | - | 56 | 7 |
|  |  |  |  |  |  |  |
| **Tumour Size** |  |  |  |  |  |  |
| **0-20mm** | 990 | 43 | 1120 | 36 | 85 | 11 |
| **21-50mm** | 1146 | 50 | 1657 | 53 | 572 | 70 |
| **>50mm** | 121 | 5 | 264 | 8 | 90 | 11 |
| **missing** | 48 | 2 | 90 | 3 | 65 | 8 |
|  |  |  |  |  |  |  |
| **Tumour Grade** |  |  |  |  |  |  |
| **1** | 76 | 3 | 49 | 1 | 21 | 2 |
| **2** | 830 | 36 | 1135 | 36 | 241 | 30 |
| **3** | 1388 | 60 | 1944 | 62 | 322 | 40 |
| **missing** | 11 | 1 | 3 | 1 | 228 | 28 |
|  |  |  |  |  |  |  |
| **Menopausal Status** |  |  |  |  |  |  |
| **Pre/ peri** | 1314 | 57 | 1660 | 53 | 506 | 62 |
| **post** | 873 | 38 | 1111 | 35 | 216 | 27 |
| **missing** | 118 | 5 | 360 | 12 | 90 | 11 |
|  |  |  |  |  |  |  |
| **BMI** |  |  |  |  |  |  |
| **Underweight (<18.5)** | 34 | 1 | 28 | 1 | 7 | 1 |
| **Healthy weight (18.5 to <25)** | 947 | 41 | 1242 | 39 | 314 | 39 |
| **Overweight (25 to <30)** | 725 | 32 | 1089 | 35 | 274 | 34 |
| **Obese (>=30)** | 489 | 21 | 764 | 24 | 216 | 26 |
| **missing** | 110 | 5 | 8 | 1 | 1 | 1 |

***Abbreviations*** *ER: estrogen receptor; PGR: progesterone receptor; HER2: human epidermal growth factor receptor; ECOG: Eastern Co-operative Oncology Group; BMI: body mass index; E: Epirubicin; C: cyclophosphamide; M: methotrexate; F: 5-fluouroucil; T: paclitaxel; G: gemcitabine*

**Table S2: NCI CTCAE version 2**

| **Toxicity Grade** | **0**  **None** | **1**  **Mild** | **2**  **Moderate** | **3**  **Severe** | **4**  **Life-threatening** |
| --- | --- | --- | --- | --- | --- |
| *Haematological* |  |  |  |  |  |
| Haemaglobin g/100ml g/l mmol/l | WNL  WNL  WNL | 10.0 – normal  100 – normal  6.2 – normal | 8.0 - 9.9  80 – 99  4.95 – 6.1 | 6.5 – 7.9  65 – 79  4.0 – 4.9 | < 6.5  < 65  <4.0 |
| Neutrophils (Granulocytes) Bands × 10^9^/L | ≥ 2.0 | 1.5 – 1.9 | 1.0 – 1.4 | 0.5 – 0.9 | < 0.5 |
| *Gastrointestinal* |  |  |  |  |  |
| Nausea | none | able to eat - reasonable intake | intake significantly decreased, but can eat | no significant intake | -- |
| Vomiting | none | 1 episode in 24 hr | 2-5 episodes in 24 hrs | 6-10 episodes in 24 hrs | >10 episodes in 24 hrs or requiring parenteral support |
| Diarrhoea | none | increase of 2-3 stools/day over pre-Rx | increase of 4-6 stools /day, or nocturnal stools | increase of 7-9 stools/day, or incontinence | increase of >10 stools/day, or grossly bloody diarrhoea, or need for parenteral support |
| Constipation | none | mild | moderate | severe | ileus > 96 hrs |
| Oral Stomatitis | none | painless ulcers, erythema, or mild soreness | painful erythema, oedema, or ulcers but can eat | painful erythema, oedema, ulcers, and cannot eat | mucosal necrosis and/or requires parenteral or enteral support |
| ***Neurological*** |  |  |  |  |  |
| Neuro-sensory | none or no change | mild paraesthesias; loss of deep tendon reflexes | mild or moderate objective sensory loss; moderate paraesthesias | severe objective sensory loss or paraesthesias that interfere with function | -- |
| ***Muscular*** |  |  |  |  |  |
| Arthralgia/Myalgia | none | mild | moderate pain limiting activities of daily living. | severe pain limiting self-care activities of daily living. |  |
| ***General*** |  |  |  |  |  |
| Infection | none | mild, no active  treatment | moderate localised  infection, requires  active treatment | severe systemic  infection, requires parenteral treatment  specify site | life-threatening  sepsis, specify site, includes febrile neutropenia |
| Fever in absence of infection | none | 37.1 – 38.0°C | 38.1 – 40.0°C | > 40°C, < 24 hrs | > 40°C, > 24hrs,  or fever with hypotension |
| Fatigue | none | mild | moderate | severe | -- |

***Abbreviations*** *WNL: within normal limits*

**Table S3: Case-Control Classification method for the 13 CRTs investigated**

| **Toxicity** | **Cases (NCI CTCAE grades)** | **N (%)** | **Controls (NCI CTCAE grades)** | **N(%)** |
| --- | --- | --- | --- | --- |
| Neutropenia | ≥3 | 1456 (25) | <3 | 4430 (75) |
| Fatigue | ≥3 | 855 (14) | <3 | 5393 (86) |
| Neuropathy | ≥2 | 1120 (28) | <2 | 2823 (72) |
| Nausea | ≥2 | 532 (9) | <2 | 5716 (91) |
| Vomiting | ≥2 | 475 (8) | <2 | 5773 (92) |
| Constipation | ≥2 | 1527 (26) | <2 | 4359 (74) |
| Diarrhoea | ≥2 | 208 (3) | <2 | 6040 (97) |
| Stomatitis | ≥2 | 155 (2) | <2 | 6093 (98) |
| Anaemia | ≥2 | 551 (14) | <2 | 3392 (86) |
| Infection | ≥2 | 2122 (34) | <2 | 4126 (66) |
| Myalgia/arthralgia | ≥2 | 1969 (50) | <2 | 1974 (50) |
| Fever | ≥1 | 135 (3) | <1 | 3808 (97) |
| Combined haematological | neutropenia ≥3 or  anaemia ≥2 or  thrombocytopenia ≥2 | 1432 (36)^a^ | neutropenia <3 and  anaemia <2 and  thrombocytopenia <2 | *2511 (64) |

^a^The figure for combined haematological is composed of patients who had data available for all three variables.

***Abbreviations*** *NCI CTCAE: National Cancer Institute Common Toxicity Criteria for Adverse Events*

**Table S4: Neutropenia and Fatigue in relation to outcome, split by different treatment components**

|  |  |  |  |  | **BCSS** | | | **RFS** | | |
| --- | --- | --- | --- | --- | --- | --- | --- | --- | --- | --- |
| **Toxicity** | **Regimen**^a^ | **Dataset** | **Trial(s)** | **N** | **N events** | **Hazard Ratio^b^ (95% CI)** | **p** | **N events** | **Hazard Ratio^b^ (95% CI)** | **p** |
| Fatigue | **E**🡪 CMF | 1 | NEAT + BR9601 | 936 | 199 | 1.48 (1.03- 2.12) | **0.03** | 269 | 1.34 (0.97-1.85) | 0.07 |
| Fatigue | **E 🡪CMF** | 2 | NEAT + BR9601 | 915 | 193 | 1.61 (1.13-2.30) | **0.009** | 263 | 1.39 (1.01-1.92) | **0.05** |
| Fatigue | **CMF** | 3 | NEAT + BR9601 | 946 | 238 | 1.10 (0.79-1.52) | 0.59 | 328 | 1.09 (0.82-1.44) | 0.56 |
| Fatigue | **EC🡪 T±G** | 4 | tAnGo + neo-tAnGo | 3312 | 669 | 0.97 (0.69-1.35) | 0.84 | 970 | 1.02 (0.77-1.34) | 0.91 |
| Fatigue | **EC 🡪T±G** | 5 | tAnGo + neo-tAnGo | 3197 | 639 | 1.18 (0.90-1.56) | 0.23 | 923 | 1.19 (0.95-1.50) | 0.13 |
| Fatigue | **T±G 🡪 EC** | 6 | neo-tAnGo | 270 | 53 | ^c^ | 0.99 | 77 | ^c^ | 0.98 |
| Fatigue | **T±G 🡪EC** | 7 | neo-tAnGo | 260 | 49 | 0.83 (0.11-6.17) | 0.85 | 72 | 0.47 (0.07-3.49) | 0.46 |
| Neutropenia | **E**🡪 CMF | 1 | NEAT + BR9601 | 807 | 162 | 0.65 (0.33-1.30) | 0.22 | 225 | 0.62 (0.35-1.10) | 0.10 |
| Neutropenia | **E 🡪CMF** | 2 | NEAT + BR9601 | 790 | 157 | 0.88 (0.50-1.57) | 0.67 | 220 | 0.78 (0.48-1.27) | 0.31 |
| Neutropenia | **CMF** | 3 | NEAT + BR9601 | 818 | 192 | 0.99 (0.67-1.50) | 0.99 | 266 | 1.00 (0.71-1.41) | 0.99 |
| Neutropenia | **EC🡪 T±G** | 4 | tAnGo + neo-tAnGo | 3312 | 669 | 0.83 (0.69-1.00) | **0.05** | 970 | 0.85 (0.73-0.99) | **0.04** |
| Neutropenia | **EC 🡪T±G** | 5 | tAnGo + neo-tAnGo | 3197 | 639 | 1.07 (0.83-1.37) | 0.62 | 923 | 1.00 (0.80-1.24) | 0.99 |
| Neutropenia | **T±G 🡪 EC** | 6 | neo-tAnGo | 270 | 53 | 0.31 (0.04-2.29) | 0.25 | 77 | 0.51 (0.16-1.65) | 0.26 |
| Neutropenia | **T±G 🡪EC** | 7 | neo-tAnGo | 260 | 49 | 0.75 (0.36-1.58) | 0.45 | 72 | 0.77 (0.42-1.44) | 0.42 |

**^a^**Patients classified into cases or controls by toxicity grade(s) recorded during treatment component in bold black

**^b^**Hazard ratios from multivariate models including trial, performance status and nodes, whilst stratifying by tumour size, tumour grade and ER status due to PH assumption.

^c^Non-calculablehazard ratio due to too few numbers

***Abbreviations****BCSS: breast cancer-specific survival; RFS: relapse-free survival; CI: confidence interval; E: Epirubicin; C: cyclophosphamide; M: methotrexate; F: 5-fluourouracil; T: paclitaxel; G: gemcitabine*

**Figure S1a & b: Trial Objectives, outcomes and treatment regimens of the contributing Clinical Trials**

**Supplementary Figure 1a**

|  | **NEAT (and BR9601)** | **tAnGo** | **Neo-tAnGo** |
| --- | --- | --- | --- |
| Ethics No. of Trial | LREC no. 96/285M | MREC no. 00/7/44 | COREC no. 04/MRE01/60 |
| Principle research question | In early breast cancer, is adjuvant chemotherapy with Epirubicin (E) following by (C) Cycolphosphamide, Methotrexate and Flurouracil (CMF) significantly superior to CMF alone in terms of DFS and OS? | In early breast cancer, does adjuvant EC following by Paclitaxel and Gemcitabine (TG) improve disease-free survival (DFS) compared with EC-T alone? | What is the role of G in a sequential neoadjuvant chemotherapy regimen of EC and T and the role of sequencing of these treatments in terms of outcome in high risk, invasive breast cancer? |
| Trial Status and No. of women recruited | Closed 07/01  Recruited 2401 | Closed 11/04  Recruited 3152 | Closed 09/07  Recruited 831 |
| Primary Endpoints | 5 year relapse free survival (RFS)  and overall survival (OS) | Primary Endpoint: DFS | Complete pathological response rates after neoadjuvant treatment. |
| Secondary Endpoints | 10 year RFS and OS; toxicity; Qualify of Life (QoL); dose intensity | 5 & 10 year OS comparisons;  10 year DFS; toxicity;dose intensity:  Serious Adverse Drug Reactions. | Clinical and radiological response after 4 & 8 cycles; RFS and OS; QoL; prognostic & predictive markers analysis; Pathological response outcome measures. |

**Figure S1b**

**tAnGo**

**Eligibility Criteria**

1. Operable breast cancer
2. Clear resection margins
3. Any nodal status

(iv) Any hormone receptorstatus

###### Stratification

(i) Country of randomising hospital: England; Scotland; Wales; Republic of Ireland, Northern Ireland

(ii) Nodal status: negative; 1-3; 4+

(iii)Age: <=50 years; >50 years

(iv)ER status: negative; weakly positive; positive

(v) Radiotherapy: no; yes

(vi)HER-2 status: +++ ; other; unmeasured

Randomisation

Arm A

4 x E 90mg/m^2^ q 3 weekly &

C: 600mg/m^2^ q3 weekly

*Followed by*

4 x Paclitaxel (T) 175mg/m^2^ q3 weekly

Arm B

4 x Epirubicin (E) 90mg/m^2^ q 3 weekly &

Cyclophosphamide (C) 600mg/m^2^ q3 weekly

*Followed by*

4 x Paclitaxel (T) 175mg/m^2^ q3 weekly

4x Gemcitabine (G) 1250mg/m^2^ day1 & 8 q3 weekly

**SURGERY**

**NEAT(**BR9601*)**

**Eligibility Criteria**

1. Early stage breast cancer
2. Tumour completely excised
3. Certain indication for chemotherapy

###### Stratification

1. Nodal status: negative; 1-3; 4+
2. Age: <=50 years; >50 years

(iii) Radiotherapy timing: none, concurrent; sequential, to be randomised into a radiotherapy sequencing trial

(iv) randomisingcentre

Randomisation

Arm A

6x CMF

Classic CMF:

Either Cyclophosphamide (C) 600mg/m^2^ q3 weekly

OR

C:100mg/m^2^ orally every day for 14 days

with

Methotrexate (M) 40mg/m^2^& 5Fluorouracil (F) 600mg/m^2^ day1 & 8

**SURGERY**

Arm B

4x Epirubicin (E): 100mg/m^2^

q3 weekly

*Followed by*

4x CMF – as per Classic CMF regimen

**Neo-tAnGo**

**Eligibility Criteria**

(i) Histologically confirmed invasive breast cancer

(ii) T2 tumour and above

(iii) Diameter >20mm

###### Stratification

(ii)Age: <=50 years; >50 years

(ii) ER status: positive; negative

(iii)Tumoursize:<=50mm; >50mm

(iv)Clinical Axillary Node

involvement; yes; no

(v)Inflammatory or locally advanced: no;yes

Randomisation

Arm A1

4 x EC

As for tango

*Followed by*

4 x T

175mg/m^2^ q2 weekly

Arm A2

4 x T

175mg/m^2^ q2 weekly

*Followed by*

4 x EC

As for tango

Arm B1

4 x EC

As for tango

*Followed by*

4 x TG

T: 175mg/m^2^ q2 weekly

G:2000mg/m^2^ q2 weekly

Arm B2

4 x T G

T: 175mg/m^2^ q2 weekly

G:2000mg/m^2^ q2 weekly

*Followed by*

4 x EC

As for tango

**SURGERY**

***Footnote***

***Modified CMF in the BR9601 trial:** Eight cycles of cyclophosphamide (750 mg per square metre), methotrexate (50 mg

per square metre), and fluorouracil (600 mg per square metre), all given intravenously on day 1 every 3 weeks.

***Epirubicin plus CMF in the BR9601 trial:** Four cycles of epirubicin (100 mg per square metre) every 3 weeks, followed

by four cycles of the modified CMF schedule.
